# *Gynostemma pentaphyllum* Tea Improves Insulin Sensitivity in Type 2 Diabetic Patients

**DOI:** 10.1155/2013/765383

**Published:** 2013-01-31

**Authors:** V. T. T. Huyen, D. V. Phan, P. Thang, N. K. Hoa, C. G. Östenson

**Affiliations:** ^1^Endocrine and Diabetes Unit, Department of Molecular Medicine and Surgery, Karolinska Institute, Karolinska University Hospital, SE 17176 Stockholm, Sweden; ^2^Hanoi Medical University, Hanoi 1000, Vietnam; ^3^Department of Endocrinology and Diabetes, National Institute of Gerontology, Hanoi 1000, Vietnam; ^4^Department of Internal Medicine, University of Manitoba, Winnipeg, MB, Canada R3T 2N2

## Abstract

*Aims*. To evaluate the effect of the traditional Vietnamese herb *Gynostemma pentaphyllum* tea on insulin sensitivity in drug-naïve type 2 diabetic patients. *Methods*. Patients received GP or placebo tea 6 g daily for four weeks and vice versa with a 2-week wash-out period. At the end of each period, a somatostatin-insulin-glucose infusion test (SIGIT) was performed to evaluate the insulin sensitivity. Fasting plasma glucose (FPG), HbA_1C_, and oral glucose tolerance tests and insulin levels were measured before, during, and after the treatment. *Results*. FPG and steady-state plasma glucose (SIGIT mean) were lower after GP treatment compared to placebo treatment (*P* < 0.001). The levels of FPG in the control group were slightly reduced to 0.2 ± 1.5 versus 1.9 ± 1.0 mmol/L in GP group (*P* < 0.001), and the effect on FPG was reversed after exchanging treatments. The glycometabolic improvements were achieved without any major change of circulating insulin levels. There were no changes in lipids, body measurements, blood pressure, and no reported hypoglycemias or acute adverse effects regarding kidney and liver parameters. *Conclusion*. The results of this study suggested that the GP tea exerted antidiabetic effect by improving insulin sensitivity.

## 1. Introduction

Type 2 diabetes mellitus (T2D) is a global health problem and is predicted to become more prevalent in the coming decades [1, 2]. Many currently available antidiabetic drugs improve the impaired insulin secretion or decreased insulin sensitivity seen in T2D. However, they exhibit a number of limitations such as side effects and high rates of secondary failure and, in the case of novel drugs, rather high treatment costs [3–5]. Thus, diabetic patients and healthcare professionals are considering complementary and alternative approaches, including the use of herbal medicine with antidiabetic properties.

Traditional herbal medicines have played a major role in the management of diabetes in Vietnam and many Asian countries for centuries [6]. *Gynostemma pentaphyllum* Makino (family Cucurbitaceae) is a perennial creeping herb growing wild in the mountain regions of Vietnam, China, and some other Asian countries. It has been used widely in Southeast Asian countries as a herbal medicine and being beneficial for the prevention and treatment of diabetes [7–9]. We have previously presented evidence that GP tea possesses antidiabetic effect, both as a single treatment and as an add-on therapy to sulfonylureas with good safety data in newly diagnosed T2D patients [10, 11]. In addition, the extract of GP has been shown to reduce both hyperglycemia and hyperlipidemia in diabetic Zucker Fatty rats [12]. Therefore, this study was aimed to evaluate the effect of GP tea on insulin sensitivity by somatostatin-insulin-glucose infusion test (SIGIT) in drug-naïve type 2 diabetic patients.

## 2. Materials and Methods

### 2.1. Medication

The medication was provided in the form of GP tea at the dose of 6 g/day (3 g/packet, twice a day). The whole plants of *Gynostemma pentaphyllum* Makino Cucurbitaceae were collected from the Hoa Binh Province, in the north of Vietnam, and identified by Professor Pham Thanh Ky, Department of Material Medica, Hanoi College of Pharmacy. The standardized GP tea was produced by a certified herbal manufacturing facility using the method described in document number VN 10907/TL, including two stages. The first stage was to confirm authenticated GP plants which were compared with the voucher specimen (HN-0152) deposited in the herbarium at the Department of Material Medica, Hanoi College of Pharmacy. The second stage was to produce the GP tea as specified. Briefly, the process included extraction of the authenticated GP plants for 2 h in boiling water and a following precipitation of impurities by adding concentrated 70% ethanol. The 70% ethanol was then removed by distillation at low pressure, and impurities were removed by filtration. Thereafter, the tea was inspected as a semifinished brown powder with typical odour of GP tea. This powder had a humidity of approximately 6.7% and could be dissolved in water into brown liquid with a sweet-bitter flavour. The tea contained flavonoids, as shown by a positive cyanidin reaction with base FeCl_3_ (5%) [13, 14], and furthermore about 18% saponins, as indicated by a positive foaming test [13, 14]. Thus, the standardization of the GP tea included confirmation of its typical odour, state, and sweet-bitter flavour, approximately 7% in humidity, and positive reaction in the flavonoid (cyanidin reaction) and saponin (foaming) tests. The placebo tea was green tea (*Camellia sinensis*), which was supplied at the same dose and was similar to the GP tea in shape and packaging. After grinding the resulting GP and placebo material into a powder to form soluble particles, the powder was packed in tin foil packets (6 × 5 cm) by an automatic packing machine. Each packet contained 3 g of powder, and 10 packets were packed in one box to be distributed to the patients. Both GP and placebo tea were easily dissolved in 60 mL water (room temperature) and taken 30 minutes prior to breakfast and dinner.

### 2.2. Patients

Sixteen patients with newly diagnosed T2D were enrolled into the study from February to September 2010 from outpatients at National Institute of Gerontology and 3 district hospitals in Hanoi. All included patients had passed through the study by November 2010. Written informed consent was obtained from all subjects before the beginning of the study. The study protocol was approved by the research ethics board at Hanoi Medical University, Hanoi, Vietnam, and the Regional Ethics Committee at the Karolinska Institutet, Stockholm, Sweden.

Inclusion criteria were (1) newly diagnosed T2D patients according to WHO criteria [15], (2) age from 40 to 70 years old, (3) antidiabetic drug naïve, (4) mean (of two) fasting plasma glucose (FPG) measurements from 7 to 11 mmol/L, and (5) glycosylated hemoglobin (HbA_1C_) from 7 to 9%.

Exclusion criteria were (1) previously pharmacological treatment for diabetes, (2) chronic complications related to type 2 diabetes, (3) smoking subjects, and (4) increased titres of GAD and IA-2 antibodies.

The evaluation at baseline included detailed medical history and clinical examination, fasting plasma glucose (FPG) and oral glucose tolerance test, (OGTT), HbA_1C_, livers and renal function tests, fasting lipid profile, and plasma insulin level. 

### 2.3. Study Designs

**Figure 1:**
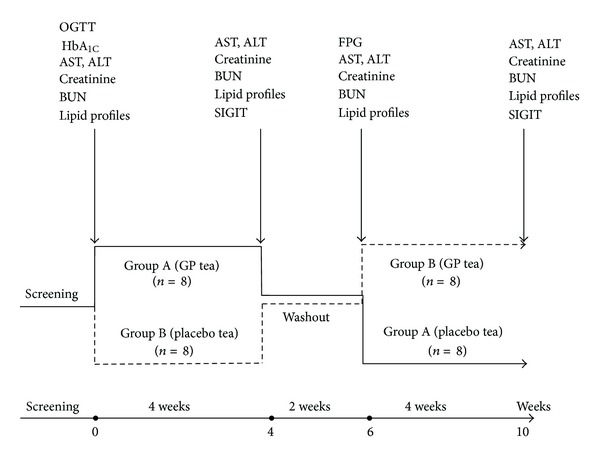
Study protocol. Lipid, kidney, and liver function tests were performed at week 0, 4, 6, and 10. Fasting plasma glucose and insulin were determined every second week. OGTTs (oral glucose tolerance tests) and HbA_1C_ were performed at baseline. SIGIT (somatostatin-insulin-glucose infusion test) was performed at the end of each treatment period.

The protocol of the study is presented in Figure 1. After screening by the main investigator, the selected patients were randomly assigned by another independent allocator by the use of numbered containers into two groups, matched by age, sex, fasting plasma glucose, and HbA_1C_: group A and group B received GP tea and placebo tea, respectively, 6 g daily in divided dose (twice a day, 30 minutes before breakfast and dinner). After two-week wash-out period of nontea therapy, the patients were switched to another four weeks of 6 g GP tea/day (group B) and placebo tea (group A). At the end of both four-week periods of treatment with GP or placebo tea (week 4 or week 10), all patients underwent a somatostatin-insulin-glucose infusion test. All subjects received comprehensive diabetes education including lifestyle and nutrition therapy. They were instructed to follow the diet as always recommended for newly diagnosed T2D patients. Carbohydrate and monounsaturated fat should comprise 60–70% of total calories. They were also instructed to walk 30 minutes a day and at least five days a week during the study. This was reinforced at each follow-up visit, and subjects were treated on an outpatient basis.

### 2.4. Somatostatin-Insulin-Glucose Infusion Test (SIGIT)

All subjects participated in SIGIT, performed at 8 am after an 8–10 h overnight fast with only tap water allowed *ad libitum*. The SIGIT, lasting 150 minutes, was conducted as described earlier [16–18]. Briefly, an i.v. cannula (Exeflon, Exelint, Los Angeles, CA, USA) was inserted into an antecubital vein for the infusion of all test substances. A second cannula was inserted into a wrist vein contralaterally to the infusion site for blood sampling. Each subject was given a 150-minute intravenous infusion of somatostatin (270 *μ*g/h; Somatosan, Wasserburger Arzneimittelwerk GmbH, Wasserburg, Germany), insulin (0.4 mU/kg/min; Actrapid HM, Novo Nordisk, Denmark), and glucose (6 mg/kg/min; in a 20% solution). Ten mL blood of each patient was added to the mixture to prevent insulin and somatostatin from absorption to glass surface. Plasma glucose and insulin were measured at 0, 30, 60, 90, 120, and 150 minutes. Somatostatin was used to suppress endogenous insulin release, thereby allowing for the estimation of sensitivity to exogenously administered insulin by measuring blood glucose value at 90, 120, and 150 minutes of the test (SIGIT mean). Since similar steady-state plasma insulin levels are achieved in all subjects, this test allows us to compare the ability of the recruited subjects to dispose of identical glucose loads under the same insulin stimulus. Therefore, the mean of several plasma glucose concentrations measured during the steady-state period from 90 to 150 minutes is a measure of efficiency of insulin-mediated glucose utilization, that is, insulin sensitivity [17, 18].

### 2.5. Biochemical and Anthropometric Analyses

Blood samples of fasting subjects were taken before, during (every second week for ten weeks), and after the treatment course for measurement of plasma glucose, HbA_1C_, liver transferase (ALT, AST), BUN, creatinine, lipid profiles, and insulin levels. Serum samples were obtained by centrifugation and stored at −20°C pending for assay. Analysis of glucose concentration of each sample was done by enzymatic colorimetric test, GOD-PAP in a glucose analyzer (Autolab Instrument, Boehringer Mannheim, Germany, wave-length Hg 546 nm). HbA_1C_ was measured with BIO-RAD D-10 (Bio-Rad Strasbourg, Schiltigheim, France). The insulin concentration was measured by insulin radioimmunoassay (RIA), using our own antibodies, human insulin as a standard, and charcoal addition to separate antibody-bound and free insulin [19]. Oral glucose tolerance tests (OGTT) (75 g glucose) were performed at baseline to confirm the diabetic diagnose. Venous blood samples were drawn before 0, 30, and 120 minutes after the glucose intake. Body weight, body mass index (BMI), waist and hip circumference, blood pressure, and registered adverse effects were noted in medical records during the visits.

### 2.6. Statistical Analysis

Results were expressed as means ± SD. Paired *t*-test was used to analyze data in the same group before and after treatment as well as the steady-state of both SIGITs. The independent sample *t*-test was used for normal distributed variables to compare differences in mean change between the treatment group and the control group (SPSS version 16.0). Statistical significance was declared at *P* < 0.05. 

## 3. Results

### 3.1. Clinical Characteristics

The baseline characteristics of the patients receiving GP treatment initially did not differ significantly from those starting with placebo and vice versa at week 6, after two weeks of washout (Table 1). There were no statistically significant differences between the groups regarding age, gender, systolic and diastolic blood pressure (SBP and DBP), body weight, BMI, waist, hip circumference, FPG, and HbA_1C_.

### 3.2. Effects of GP Tea on Glucose Regulation

**Figure 2:**
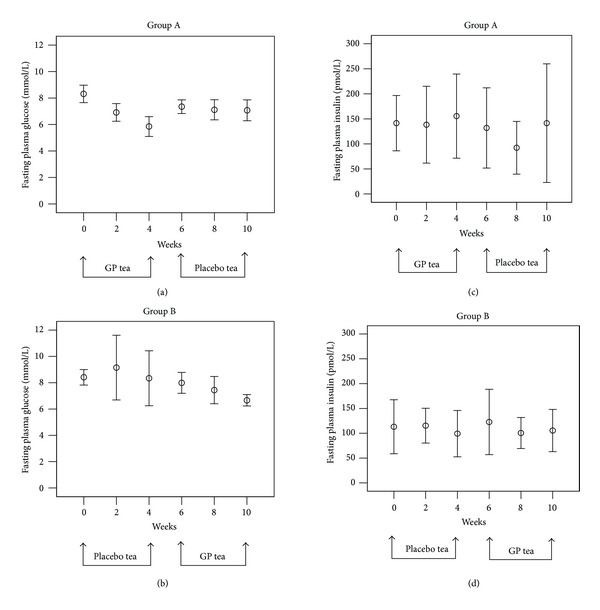
Fasting plasma glucose (mmol/L) and insulin (pmol/L) levels in Group A ((a) and (c)) and Group B ((b) and (d)). Means ± SD (*n* = 8 in each group).

Treatment with GP tea compared to placebo induced no differences in fasting plasma insulin (FPI) but resulted in lower FPG (*P* < 0.001; Table 2). Following the treatment with GP tea, the FPG decreased to1.9 ± 1.0 mmol/L, while the levels of FPG in the placebo treatment were not changed, 0.2 ± 1.5 mmol/L, *P* < 0.001. The effects were reversed after exchanging treatments (Figure 2), and the glycometabolic improvement was achieved without any major change of circulating insulin levels (Table 2, Figure 2).

The mean steady-state plasma insulin levels and plasma glucose responses of the two groups during SIGIT study are given in Table 2. The steady-state plasma insulin (SSPI) response clearly indicates that similar plasma levels of exogenous insulin were attained as a result of the infusion in all subjects. In contrast, the mean steady-state plasma glucose (SSPG) responses were markedly decreased after the GP tea treatment (*P* < 0.01), indicating improved insulin sensitivity.

### 3.3. Change in Body Weight and Other Parameters

There were no significant differences within or between groups regarding changes in serum triglyceride, total cholesterol, and HDL and LDL cholesterol levels. Similarly, no significant changes in the plasma levels of AST, ALT, creatinine, and BUN were detected during treatment (data not shown). Neither the GP tea-treated nor placebo-treated groups experienced any acute adverse effect such as gastrointestinal, diarrhea, and hypoglycemic symptoms during the research period, and all patients were compliant with the treatment protocol and completed the study.

## 4. Discussion

The main findings of this study were that GP tea exerted a significant antidiabetic effect and that this was accounted for by enhanced insulin sensitivity.

The placebo group and the GP group did not differ in baseline characteristics and diabetic parameters (drug-naïve T2D, HbA_1C_, and FPG). The GP tea was mainly responsible for the reduction of the glucose levels because all patients received similar diet and exercise therapies, with minor effect on FPG in the control group. The major antidiabetic role of GP tea was also proven by the reduction of the FPG and the glucose responses to insulin during SIGIT after GP treatment with reversed effect after switching to placebo treatment, whereas the plasma insulin during SIGIT did not change after GP tea treatment in our study population.

We assessed insulin sensitivity by SIGIT, during which the endogenous production of insulin and C-peptide is inhibited by a simultaneous infusion of somatostatin and insulin. Serum glucose levels during the last part of a SIGIT are used as a measure of the sensitivity to insulin in each individual. Achievement of a continuous suppression of plasma C-peptide levels during SIGIT guarantees that glucose disappearance is governed by the infused and not by endogenously secreted insulin. Insulin sensitivity measured with SIGIT is significantly correlated with *M* values obtained during hyperinsulinemic euglycemic clamp, which is a “gold standard” in measuring insulin sensitivity [20, 21]. In a previous study, endogenous C-peptide concentrations, reflecting insulin secretion during SIGIT, were almost entirely abolished by somatostatin [18]. In addition, in our previous study we showed that the glycometabolic improvement was achieved without any major change of circulating insulin and C-peptide levels [11]. Thus, our present results are in agreement with our previous findings by GP tea [10, 11] and indicate that the decrease in blood glucose levels is explained by the improvement in insulin sensitivity. According to our previous studies in twelve weeks, HOMA-IR decreased significantly after GP tea treatment [10]. This observation, combined with the present results, suggests that GP tea provides improved glycemic control via a mechanism that does not involve stimulation of insulin release and thus does not place any additional burden on defective *β*-cells. An effect by GP tea on insulin sensitivity may be accounted for by suppression of PTP-1B activity by dammarane compounds in the GP tea [22]. PTP-1B is present in liver and skeletal muscle and has been shown to negatively modulate insulin's action on hepatic glucose metabolism through tyrosine dephosphorylation of the insulin receptor and/or insulin receptor substrates. Interestingly, a recent experimental study demonstrated that an ethanol extract of GP, produced in Vietnam, inhibited protein tyrosine phosphatase 1B activity, which may lead to enhanced insulin sensitivity and thereby improved glucose tolerance [22].

In this study, green tea (*Camellia sinensis*) was used as a placebo compound. Green tea was reported to induce antihyperglycemic effect in mice and streptozotocin-diabetic rats [23, 24], but there is little evidence that it improves glycemic control substantially in human type 2 diabetes [24, 25].

In the GP group, the liver and renal function tests were normal during the study period. This finding was in accordance with our previous results [10, 11] and was evidence for the biosecurity of using GP tea as an antihyperglycemic treatment. This notion is supported also by a study in rats where no signs of chronic toxicity were found after 6-month administration of rather high GP extract doses (up to 0.75 g/kg per day) [26]. During the study, no patient experienced symptoms of hypoglycemia.

This trial only enrolled a modest number of patients; larger and longer trials are needed to assess, with higher accuracy, the prevalence of possible adverse effects. In addition, further research is needed to determine the durability of GP extract's antidiabetic effect, as well as effects on patient-reported outcomes, morbidity, and mortality.

## 5. Conclusion

In conclusion, the results of this placebo-controlled crossover study suggested that the GP tea exerted antidiabetic effect by improving insulin sensitivity.

## Figures and Tables

**Table 1:** Clinical characteristics and laboratory findings of the patients.

	Group A (GP-placebo tea)		Group B (placebo tea-GP)	
	Baseline	Week 6	Baseline	Week 6
*n*	8	8	8	8
Age (years)	58.8 ± 5.9	58.8 ± 5.9	58.1 ± 6.6	58.1 ± 6.6
Sex (male : female)	5 : 3	5 : 3	5 : 3	5 : 3
Body weight (kg)	58.6 ± 5.3	58.8 ± 5.4	56.8 ± 4.9	57.1 ± 5.0
BMI (kg/m^2^)	23.3 ± 1.9	23.4 ± 2.0	22.6 ± 1.8	22.7 ± 1.9
Waist (cm)	85.8 ± 6.2	85.4 ± 5.8	81.0 ± 6.1	81.9 ± 6.0
Hip (cm)	94.4 ± 3.5	94.1 ± 3.3	91.8 ± 4.2	91.6 ± 4.0
Systolic blood pressure (mmHg)	119.4 ± 10.2	119.4 ± 11.5	118.2 ± 8.3	118.5 ± 7.9
Diastolic blood pressure (mmHg)	74.4 ± 5.0	74.4 ± 4.9	76.3 ± 4.2	76.3 ± 5.1
Fasting plasma glucose (mmol/L)	8.3 ± 0.8	7.4 ± 0.6	8.4 ± 0.7	8.0 ± 1.0
Fasting plasma insulin (pmol/L)	140.98 ± 38.2	132.0 ± 109.2	113.2 ± 65.3	122.9 ± 78.5
HbA_1C_ (%)	8.1 ± 0.9		8.1 ± 0.7	
Cholesterol (mmol/L)	5.4 ± 0.8	5.4 ± 1.0	5.0 ± 0.9	5.4 ± 1.2^a^
Triglyceride (mmol/L)	2.2 ± 1.5	2.1 ± 0.9	2.1 ± 1.1	2.7 ± 1.3^a^
HDL-cholesterol (mmol/L)	1.5 ± 1.0	1.2 ± 0.2	1.1 ± 0.2	1.1 ± 0.2^a^
LDL-cholesterol (mmol/L)	3.3 ± 1.2	3.3 ± 1.0	3.0 ± 0.7	3.0 ± 0.8^a^

Results are means ± SD of sixteen patients. ^a^ *n* = 7, after removal of one patient with an extreme triglyceride value (12 mmol/L).

**Table 2:** Plasma glucose, insulin levels, and SIGIT mean in two kinds of treatment.

	GP treatment	Placebo treatment	*P* value
FPG before treatment (mmol/L)	8.2 ± 0.9	7.9 ± 0.8	0.385
FPG after treatment (mmol/L)	6.3 ± 0.8	7.7 ± 1.9	0.039
Change in FPG (mmol/L)	−1.9 ± 1.0	−0.2 ± 1.5	<0.001
SSPG (SIGIT mean) (mmol/L)	12.5 ± 3.2	16.2 ± 4.1	<0.001
FPI before treatment (pmol/L)	123.6 ± 59.7	122.2 ± 79.9	0.971
FPI after treatment (pmol/L)	130.6 ± 81.3	120.1 ± 106.3	0.773
Change in FPI (pmol/L)	−0.23 ± 7.8	−0.3 ± 9.8	0.984
SSPI (pmol/L)	223.6 ± 91.0	218.1 ± 104.9	0.696

Results are means ± SD of sixteen patients in each treatment. FPG: fasting plasma glucose, FPI: fasting plasma insulin, SSPG: steady-state plasma glucose, and SSPI: steady-state plasma insulin.
